# Synchronous Bone Metastasis From Multiple Myeloma and Prostate Adenocarcinoma as Initial Presentation of Coexistent Malignancies

**DOI:** 10.3389/fonc.2018.00137

**Published:** 2018-04-30

**Authors:** Diego Andres Adrianzen Herrera, Shlomit Goldberg-Stein, Alexander Sankin, Judy Sarungbam, Janaki Sharma, Benjamin A. Gartrell

**Affiliations:** ^1^Department of Oncology, Albert Einstein College of Medicine/Montefiore Medical Center, Bronx, NY, United States; ^2^Department of Radiology, Albert Einstein College of Medicine/Montefiore Medical Center, Bronx, NY, United States; ^3^Department of Urology, Albert Einstein College of Medicine/Montefiore Medical Center, Bronx, NY, United States; ^4^Department of Pathology, Albert Einstein College of Medicine/Montefiore Medical Center, Bronx, NY, United States

**Keywords:** bone metastasis, radiographic imaging, synchronous malignancies, multiple myeloma, prostate cancer

## Abstract

The radiographic appearance of bone metastases is usually determined by tumor histology and can be osteolytic, osteoblastic, or mixed. We present a patient with coexistent bone metastasis from multiple myeloma and prostate adenocarcinoma who exhibited synchronous bone involvement of both histologies within the same bone lesion, a rare phenomenon that has not been previously reported and led to atypical radiographic findings. The radiograph of a 71-year-old man with thigh swelling and pain demonstrated a lytic femoral lesion. Magnetic resonance imaging (MRI) confirmed a destructive process, but showed coexistent metaphyseal sclerosis. Multiple myeloma was suspected by demonstration of monoclonal gammopathy and confirmed by computed tomography (CT)-guided biopsy. Incidentally, CT demonstrated areas of sclerosis corresponding to T_2_ hypointensity on MRI. Further studies revealed osteoblastic spinal metastasis, prostate enhancement on CT and prostate-specific antigen (PSA) level of 90 ng/mL, concerning for concomitant prostate neoplasm. After endoprosthetic reconstruction, pathology of the femur identified both plasma cell neoplasm and metastatic prostate adenocarcinoma. An association between prostate cancer and multiple myeloma is hypothesized due to tumor microenvironment similarities and possible common genetic variations, however, coexisting bone metastases have never been reported. This unusual finding explains the discrepant imaging features in our patient and is evidenced that certain clinical situations merit contemplation of atypical presentations of common malignancies even if this leads to additional testing.

## Introduction

Metastases are the most common type of malignancy affecting the bones (1). Bone metastases are classified as osteoblastic, osteolytic, or mixed depending on their radiographic appearance, which in turn is frequently determined by the underlying tumor histology. Osteolytic lesions are radiographically lucent and are characterized by osteoclast-mediated bone destruction. These lesions are commonly present in multiple myeloma, renal cell carcinoma, thyroid cancer, and melanoma. Osteoblastic lesions are radiographically dense and sclerotic, and are typically present in prostate adenocarcinoma, small cell lung cancer, Hodgkin’s lymphoma, and carcinoid tumors. Mixed lesions are both osteolytic and osteoblastic and are typical of gastrointestinal tumors or squamous cell carcinomas (2).

Coexistent malignancies are usually associated with common genetic, infectious, environmental, or occupational factors (3), but some synchronous malignancies may be coincidental. A potential association between prostate adenocarcinoma and multiple myeloma has been proposed but not confirmed (4, 5), with only a few reported cases. We present a patient with atypical radiographic findings caused by synchronous occurrence of multiple myeloma and prostate adenocarcinoma metastasis within the same bone lesion. To our knowledge, this is the first case reported of this unusual phenomenom which in retrospect highlights the utility of proper radiographic imaging assessment and interpretation for cancer patients.

## Background

**Figure 1:**
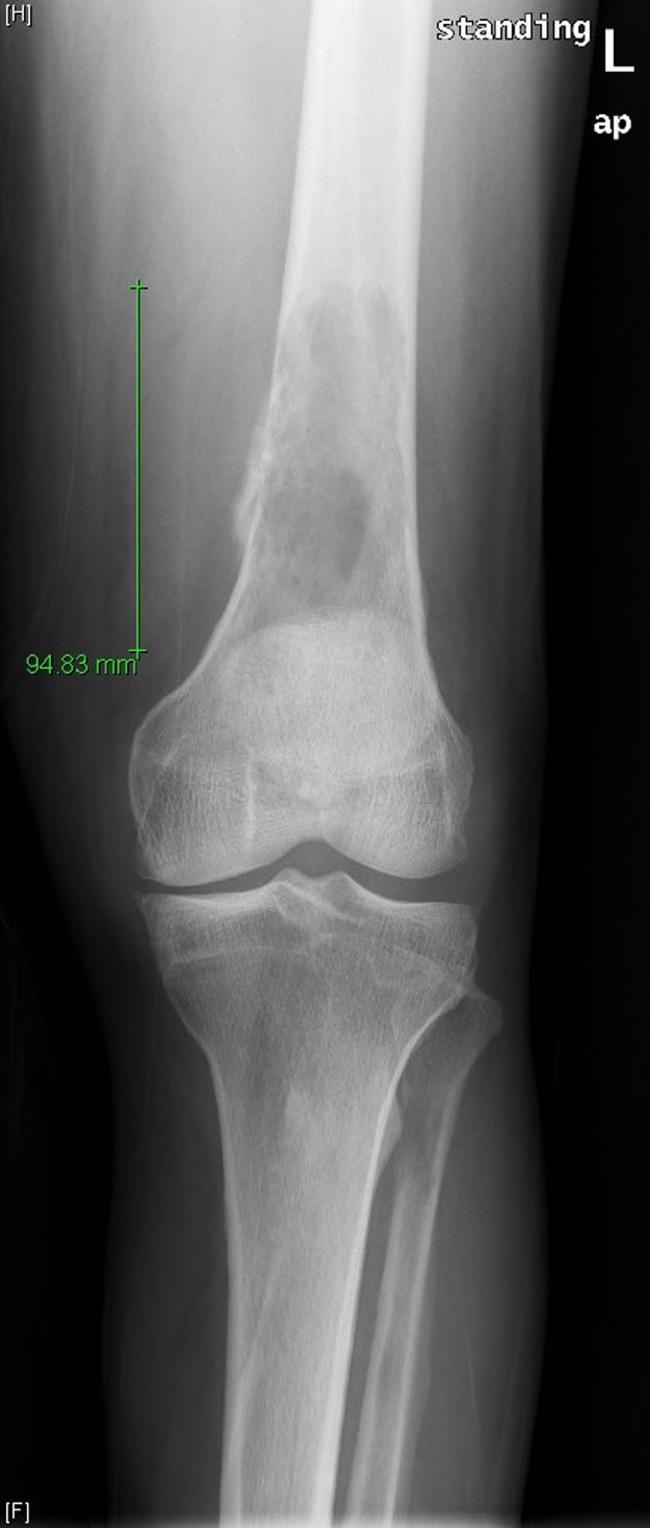
Frontal radiograph of the left femur demonstrates a large lytic lesion of the distal diaphysis with wide zone of transition and aggressive-appearing periosteal reaction at the medial margin.

**Figure 2:**
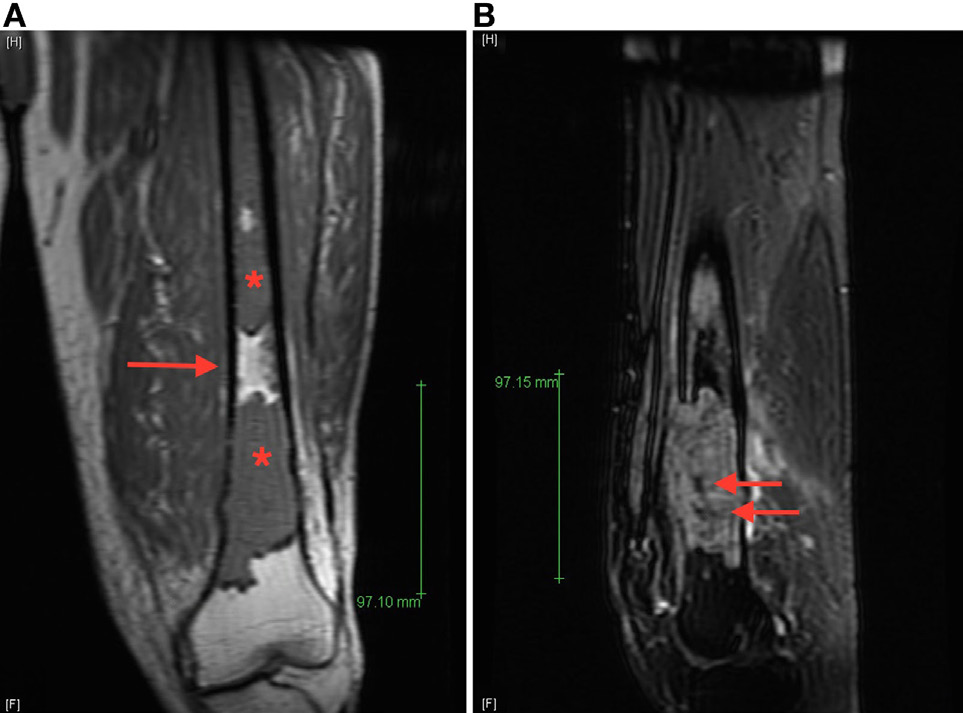
**(A)** Coronal T_1_ magnetic resonance imaging (MRI) image of the left femur demonstrates T_1_ hypointense lesions (starts) with normal intervening bone marrow (arrow), **(B)** sagittal T_2_ fat-saturated MRI image of the dominant distal femur lesion demonstrated intermediate T_2_ hyperintensity with areas of marked low signal intensity centrally (arrows), suggestive of areas of sclerosis not appreciated radiographically.

A 71-year-old man was evaluated in orthopedics clinic for progressive left thigh swelling and pain leading to impaired ambulation for 3 months. Radiographs demonstrated a large lytic lesion of the distal femur with cortical destruction (Figure 1). Urgent evaluation with magnetic resonance imaging (MRI) confirmed a 10 cm destructive distal metadiaphyseal lesion in the left femoral diaphysis as well as coexistent central areas of T_2_ hypointensity suggestive of metaphyseal sclerosis which were not appreciated radiographically (Figure 2). Multiple myeloma was clinically suspected on the basis of normocytic anemia, increased serum creatinine, and elevated globulin levels. Serum protein electrophoresis showed an IgA-kappa monoclonal M-spike and percutaneous computed tomography (CT)-guided biopsy of the femoral lesion was consistent with plasma cell neoplasm affecting the bone.

**Figure 3:**
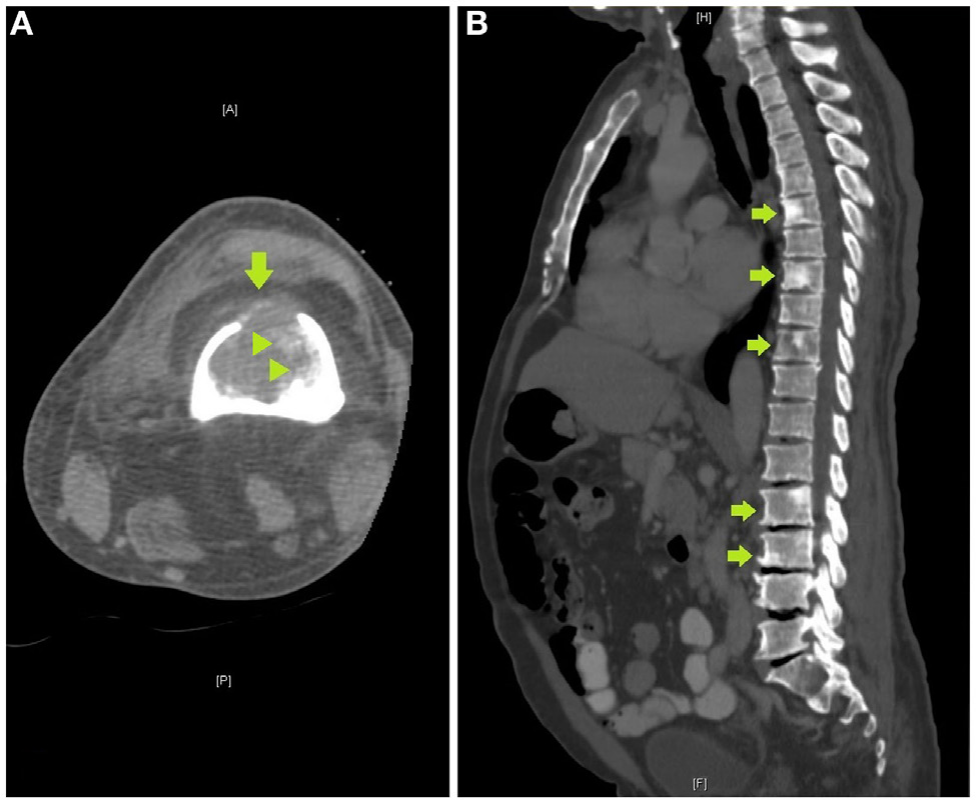
**(A)** Axial computed tomography (CT) image of the femur obtained to localize the lesion for CT-guided biopsy demonstrates a destructive lytic lesion with cortical breakthrough anteriorly (arrow) and areas of bone sclerosis (arrowheads), **(B)** sagittal image of a CT of the chest, abdomen, and pelvis demonstrates multiple sclerotic lesions in the spine, including within T6, T8, T10, L2, and L3 vertebrae (arrows).

Review of the axial CT images of the femur obtained to localize the lesion for biopsy demonstrated a predominantly lytic lesion and also small areas of sclerosis (Figure 3A) corresponding to the areas of T_2_ hypointensity on MRI (Figure 2B). Given the atypical MRI findings of sclerotic appearance on T_2_, a CT of the chest, abdomen, and pelvis was performed and demonstrated diffuse osteoblastic metastatic lesions, including multiple sclerotic lesions throughout the spine (Figure 3B). Pelvic images also demonstrated asymmetric enhancement of the left anterior prostate and prominent retroperitoneal and pelvic lymph nodes, which were concerning for concomitant metastatic prostate neoplasm. Serum prostate-specific antigen (PSA) level was 90 ng/mL.

**Figure 4:**
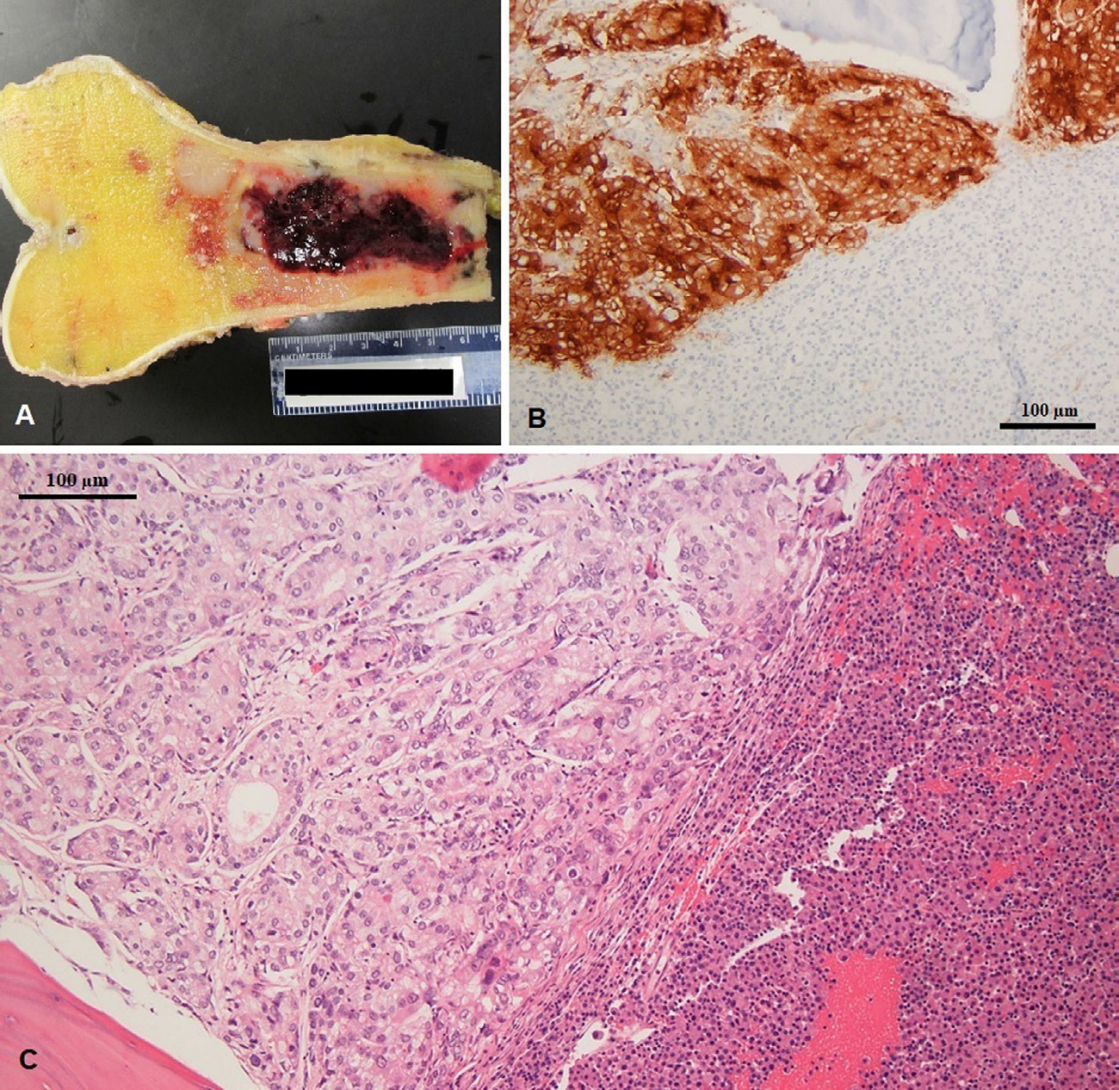
**(A)** Excised left femur with hemorrhagic tumor involving the diaphysis (gross specimen), **(B)** prostatic specific membrane antigen expression in prostate adenocarcinoma (left) and negative in multiple myeloma (right), and **(C)** coexisting metastatic prostate adenocarcinoma and multiple myeloma in same histologic section; note the gland forming adenocarcinoma (left) and sheets of plasma cell infiltration (right).

The patient finally underwent distal femur resection with endoprosthetic reconstruction. Pathology examination of the distal femur identified both plasma cell neoplasm and metastatic adenocarcinoma of prostate origin within the same metastatic lesion (Figure 4). The final diagnoses were stage IV hormone-naïve prostate adenocarcinoma and ISS stage III IgA-kappa multiple myeloma. Treatment was started with bicalutamide and leuprolide for androgen deprivation therapy (ADT), which achieved PSA suppression after 3 months. Myeloma was simultaneously treated with five 28-day cycles of cyclophosphamide, bortezomib, and dexamethasone followed by autologous hematopoietic stem cell transplantation with melphalan conditioning, with the patient achieving a very good partial response (VGPR). Currently, at 2 years of follow-up, he remains on leuprolide for ADT with continued suppression of PSA and is on maintenance lenalidomide with persistent VGPR from myeloma. No bone-specific events have occurred and his bone pain is well controlled.

## Discussion

The incidence of simultaneous diagnosis of prostate cancer and lymphoid malignancies is reported to be approximately 1.2% (6), but synchronous occurrence of prostate adenocarcinoma and multiple myeloma is only reported in a few cases (3, 4, 6). Coexistence of plasma cell neoplasm and metastatic prostate carcinoma in the bone marrow has been described (6, 7), but synchronous skeletal metastases from these malignancies affecting the same bone lesion as was found in our patient has never been reported.

An association between prostate cancer and multiple myeloma has been hypothesized based on similarities in the tumor microenvironment of both malignancies and possible shared biological pathways leading to co-stimulatory mechanisms (4). These include growth factors and antiapoptotic cytokines, such as interleukin-6 (8) and insulin-like growth factor 1 (9), which are commonly released by myeloma cells and might play a role in prostatic proliferation by activation of the RAS–MAPK pathway; or stromal-derived factor 1, a common chemokine which causes selective adhesion to bone tissue in myeloma cells, but can also participate in the pathogenesis of prostatic bone metastasis (4). Additionally, myeloma-induced immunodeficiency might be a factor contributing to more aggressive phenotype and accelerated progression of prostate cancer (6). Furthermore, common genetic variations have been proposed based on cancer registry familial studies which showed increased relative risk of multiple myeloma in families with higher number of prostate cancer cases and families whose members with prostate cancer were diagnosed at younger age (5). Although these mechanisms are not yet clear, the relationship between multiple myeloma and prostate cancer should be further explored.

Variable or mixed radiographic patterns are well documented with certain malignancies like breast, bladder, or gastrointestinal tumors (2, 10), but our patient exhibited atypical imaging inconsistent with his cancer diagnosis. As such, the sclerotic changes seen on imaging were not in line with the initial diagnosis of multiple myeloma given that osteoblastic lesions are extremely unusual in this condition with only a few reported cases (11), as would be expected due to the underlying osteoclast activation which drives skeletal metastasis in multiple myeloma. Similarly, even though metastasis mechanisms in prostate cancer include local, hematogenous, and lymphatic spread which can lead to atypical presentations (12), osteolytic lesions occur in less than 5% of metastatic prostate cancer (3), and a large destructive lesions with cortical involvement which was not consistent with a diagnosis of prostate adenocarcinoma. Thus, in our case, the radiographic findings led us to suspect that two malignancies were presenting synchronously, despite initial pathological confirmation of one of them. After surgical excision, the unusual finding of prostate adenocarcinoma and multiple myeloma within the same bone specimen explained the discrepant imaging features of the distal femur lesion, having features of both osteolytic and osteoblastic (marked hypointensity on T_2_ and areas of sclerosis on CT) lesion.

## Concluding Remarks

Understanding and distinguishing the characteristic appearance of multiple myeloma and prostate cancer skeletal metastasis guided appropriate diagnostic testing and ultimately allowed for proper treatment in this patient who continues to respond well to cancer therapy to this day. Although cost-conscious avoidance of excessive testing is usually the preferred approach in oncological clinical practice, specific clinical situations, such as discordant imaging and atypical presentations of common malignancies, warrant consideration of unusual presentations, even if it requires additional testing, and especially, when it may impact treatment.

## Ethics Statement

This work was exempted from ethics approval by Montefiore Einstein Center for Bioethics because it constitutes a purely descriptive case report. The report does not involve any risk to the participating patient, including no foreseeable risk, harm, or discomfort. Written informed consent was granted by the patient reported in this work for the publication of this case report and radiographic images.

## Author Contributions

DH and BG conceived the presentation of this case and summarized the findings. JSh reviewed all clinical information regarding multiple myeloma. AS reviewed all clinical information regarding prostate cancer. SG-S performed the review and description of radiographic studies. JSa verified and described the pathological findings. All authors discussed the results and contributed to the final manuscript.

## Conflict of Interest Statement

The authors declare that the research was conducted in the absence of any commercial or financial relationships that could be construed as a potential conflict of interest.
